# The Anterior Globus Pallidus Externus of Basal Ganglia as Primarily a Limbic and Associative Territory

**DOI:** 10.7759/cureus.11846

**Published:** 2020-12-02

**Authors:** Paul Saad, Karina S Shendrik, Paul J Karroum, Heela Azizi, Ayodeji Jolayemi

**Affiliations:** 1 Psychiatry, American University of Antigua College of Medicine, Coolidge, ATG; 2 Psychiatry, Interfaith Medical Center, Brooklyn, USA

**Keywords:** basal ganglia, globus pallidus, associative territory, behavioral symptoms, limbic territory, psychosis, cognition, motor dysfunction

## Abstract

There have been an increasing number of functions attributed to the basal ganglia, such as cognitive, emotional, and motor functions. As a result, there is a growing interest to localize these functions to different subregions of the basal ganglia. Most research on localization has been conducted on animals. The experiments subdivide the basal ganglia regions into motor, limbic, and associative functioning areas. There are sparse reports on the localization of functions in humans. This paper attempts to provide such localization of function with a focus on the globus pallidus externus of the basal ganglia. We present the case of a young man who had impairment in mixed cognitive, perceptual, and mood disturbances. No significant motor symptoms were noted in the patient. Brain imaging demonstrated dense bilateral calcifications in the basal ganglia, bilaterally localizing to the anterior region of the globus pallidus externus. We discuss our findings in light of recent studies that imply that isolated pathology in the anterior region of the globus pallidus externus may be associated with behavioral, mood, and cognitive disturbance without motor symptoms.

## Introduction

The structures and circuitry of the basal nuclei are located in various regions of the brain. They consist of subnuclei embedded deep within the brain’s hemispheres (caudate-putamen or striatum and globus pallidus [GP]), as well as related nuclei located in the mesencephalon (substantia nigra), diencephalon (subthalamic nucleus), and pons (pedunculopontine nucleus) [1]. The components of the basal ganglia can be broadly categorized into three groups: input nuclei, output nuclei, and intrinsic nuclei. The input nuclei refer to those structures that receive incoming information mainly from the cerebral cortex, thalamus, and substantia nigra. They consist of the caudate nucleus, the putamen, and the nucleus accumbens. These structures receive afferent cortical and thalamic information to be further processed within the basal ganglia system. The output nuclei refer to those structures that send information to the thalamus. They consist of the globus pallidus internus (GPi) and the substantia nigra pars reticulata. These structures mainly project to the ventral nuclei of the thalamus, which, in turn, project to the cerebral cortex (mainly the frontal lobe). Located between the input and output nuclei are the intrinsic nuclei, which are responsible for the relay of information between these two regions. The intrinsic nuclei consist of the globus pallidus externus (GPe) and the substantia nigra pars compacta.

Although it was originally believed that the basal ganglia were best known for its relevance to motor control, in the last few decades that concept has changed dramatically [1]. The basal ganglia now encompass a more complex set of functions that includes mediation of goal-directed behaviors, emotion, motivation, and cognition [2]. Anatomical tract tracing studies have been developed, revealing specific interactions between the GP and different cortices, thus determining functions. The anteroventral GP communicates with the prefrontal and orbitofrontal cortices, which are involved in motivational control. The anterodorsal GP communicates with the lateral prefrontal cortex, which is involved in cognitive control. The posterior GP communicates with the frontal motor cortex, which is involved in action control. Thus functioning of the basal ganglia has been thought to correlate with these connections of the nuclei regions to motor, associative, or limbic areas [3].

Although human case reports have presented cases of isolated non-motor symptoms with gross basal ganglia abnormalities [3], most of the findings of regions pertaining to the basal ganglia have been described by animal experiments. When it comes to segregation of basal ganglia subregions to motor, associative, and limbic areas, most of the research involved animal subjects. The animals used were, mostly rodents, fish, and rats, whereas the number of non-human primates used in research is <1%. The impact of studies using non-human primates on human health, particularly when it comes to our understanding of the brain, is significant [3]. They may lead to generalized correlations for human health, especially with current evidence suggesting that the overall structural and functional architectures of these regions are similar in humans and non-human primates. The generalized similarities between the primate and human GPe are the focus of this paper.

The identification of neuronal circuits of the GPe has been investigated using retrograde neurotropic viruses. Kelly and Strick describe injections of a virus into the primary motor cortex, resulting in neuronal labeling in the posteroventral portion of the GPe [4]. In contrast, viral injections into the prefrontal cortex result in neuronal labeling in the anterodorsal portion of the GPe, which indicates that this subportion corresponds to the associative territory [3]. The anteroventral aspect of the GP is shown to receive main inputs from the anteroventral striatum, which suggests that this portion of the GPe corresponds to the limbic territory [5]. Because no studies to date have injected viral injections into the limbic cortex, the location of the limbic territory within the GPe remains unclear.

Although these findings can be generalized to humans, there remains a need for human findings in keeping with observations in animals, especially non-human primates. The closest to such findings in humans was a recent study by Cacciola et al. using diffusion MRI and tractography [6]. Their results localized GP clusters identified as “sensorimotor”, “associative”, and “other”. The results also challenged the traditional model of the basal ganglia network by showing the possible existence, in the human brain, of cortico-pallidal, cortico-nigral projections [6]. In addition, there has been an absence of clinical evidence that investigates these findings and potentially provides evidence for or against this model by Cacciola against traditional models. Such a human finding may also resolve the question of whether the limbic territory of the GPe localizes to the anterior region as it is presumed to in monkeys.

We therefore present the case of a young man who had impairment in mixed cognitive, perceptual, and mood disturbances. No significant motor symptoms were noted in the patient. Brain imaging demonstrated dense bilateral calcifications in the basal ganglia, bilaterally localizing to the anterior region of the GPe. We discuss our findings and the possible role of the anterior GPe as being a territory for connections with the associative cortex and limbic cortex.

## Case presentation

We present a case of a 35-year-old bilingual male, single, unemployed, domiciled at a supervised residence, with a reported past psychiatric diagnosis of unspecified schizophrenia. He was brought to the ED by ambulance for bizarre behavior in the context of non-adherence with treatment. Mental Status Exam in the ED revealed a young adult of average height and build, with poor hygiene and grooming. He had poor eye contact and demonstrated psychomotor agitation, with unpredictable behavior. He described his mood as “good”, though his affect was irritable, labile, inappropriate, and overly reactive. He exhibited both concrete thinking and thought blocking at times along with associative loosening and tangentiality. His thought content was devoid of suicidal or homicidal ideations, intents, and plans. However, paranoid and persecutory delusions were evident during the interview, as demonstrated by verbal mistrust in staff and law enforcement.

He was very uncooperative and irritable during the interview, especially when questioned regarding impairment in his recollection of immediate events and remote events were presented. He was unable to sustain communication with the interviewer due to irritability and attempted to end the interview on multiple occasions by using his native tongue, although he was proficient in the English language as demonstrated later in the interview. He also exhibited both poor insight and reality testing, frequently reporting that nothing was wrong with him and that he did not need any medications. He denied any perceptual disturbances though he appeared internally preoccupied, often staring aimlessly at the wall and mumbling to self. He exhibited some paranoid ideations elicited by his request to read the interviewer’s notes before proceeding with the interview. He said he wanted to confirm if what the interviewer had written down was true. He later became verbally aggressive towards the interviewer and staff members, demonstrating poor impulse control. A chart review showed the patient has had multiple visits to the psychiatric ED and inpatient hospitalizations with similar presentations within one year and was diagnosed with unspecified schizophrenia. He later reported that he was born in the United States (US) but taken back to his native country as an infant. He returned to the US at age 23 for further education but faced difficulties due to financial constraints. Shortly thereafter, he stated that he became afraid, thinking something was going to happen to him. Subsequently, he was hospitalized for a week. This was his first psychotic episode 12 years ago. He later developed the same feelings of paranoia and was again hospitalized at another hospital, this time for three months. He was later connected to outpatient care, which he adhered to until a year ago. This was his sixth hospital admission in 15 months. Most episodes of disorganization resolved within three to four days. There was no other reported significant past medical history. The patient was informed of medical management plan, and consent was received from the patient to undergo voluntary hospitalization for further assessment and stabilization.

A mini-mental state examination score was 21 out of 30, indicating mild cognitive impairment. On orientation, the patient scored 10 out of 10 points. On registration, he scored 3 out of 3 points; however, he needed four attempts to recall the unrelated objects. The patient scored 1 out of 5 points in attention and only scored 1 out of 3 points in delayed recall. When the patient’s competency in language was tested, he failed to follow directions to use both hands to fold the paper in half before placing it on the floor. He also failed to write a sentence containing a subject and a verb and failed to copy the five-sided figure, receiving 6 out of 9 in language. The patient was cooperative throughout the examination and made efforts to follow directions when instructed.

Neurological examination showed intact cranial nerves except for a notable lack of convergence. The patient demonstrated cognitive distortions in the absence of motor symptoms commonly found in basal ganglia pathology such as parkinsonism, hemiballism, and chorea like movements. Our patient showed no signs of gait abnormalities, loss of balance, or upper and lower motor neuron lesion signs.

**Figure 1:**
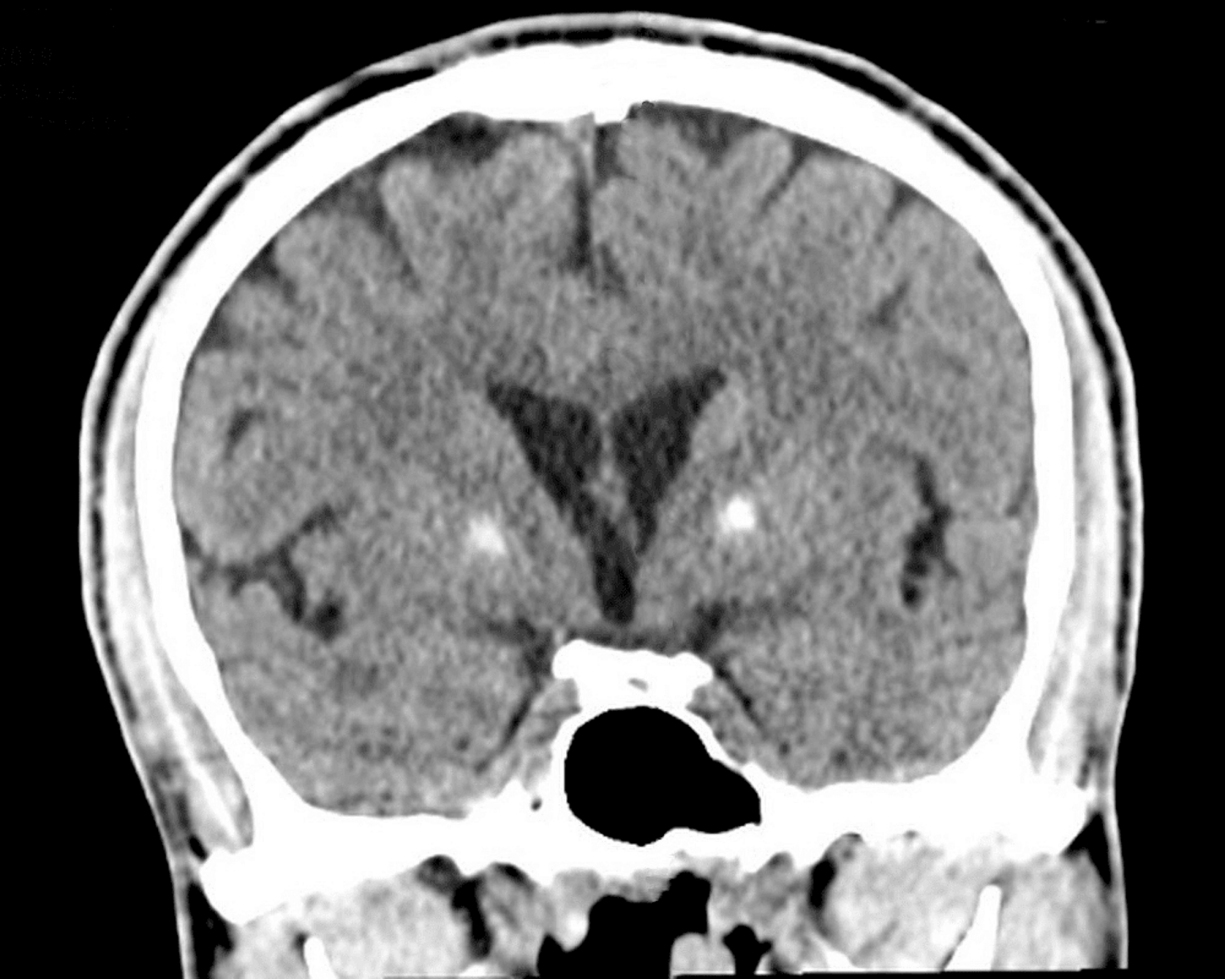
A coronal plane CT scan of the patient's brain showing calcification limited to the anterior region of the globus pallidus externus.

**Figure 2:**
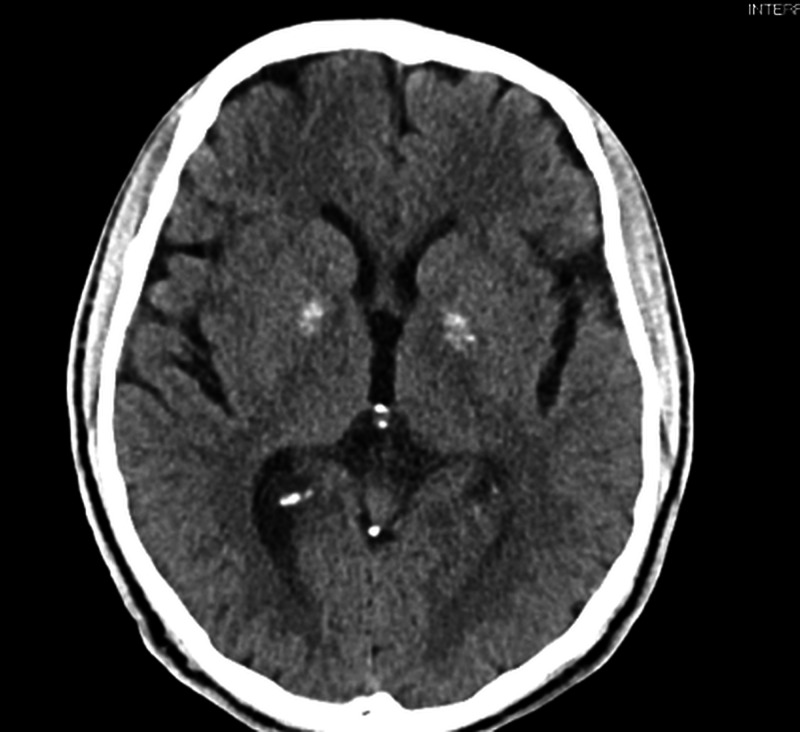
A transverse plane CT scan of the patient’s brain showing calcification limited to the anterior portion of the globus pallidus externus.

A non-contrast CT scan of the head showed dense bilateral basal ganglia calcifications, bitemporal volume loss, and cerebral atrophy (Figure 1). A closer examination revealing the pathology of calcification of the basal ganglia was limited to the anterior region of the GPe calcification (Figure 2). The posterior region of the GPe was not involved, and there was no evidence of acute intracranial pathology. Metabolic processes such as hypoparathyroidism, pseudohypoparathyroidism, and hyperparathyroidism were ruled out given normal thyroid and parathyroid functions. Urine toxicology, complete blood count including hemoglobin, and comprehensive metabolic panel were all found to be within normal limits.

The patient’s medication regimen consisted of risperidone 2 mg PO (per os) twice daily for psychosis, valproic acid 500 mg PO twice daily for mood stability and aggressivity, diphenhydramine 50 mg PO at bedtime as needed for sleep, and medication for agitation as needed. During the first two days of his hospital stay, the patient remained uncooperative, hyperactive, and irritable, and consistently demonstrated poor impulse control. Collateral information obtained from his case manager and residence revealed that our patient has a consistent pattern of aggressive behavior towards staff, workers, and other residents. His case manager reported that the patient was stable on haloperidol before becoming non-compliant. His medications were then adjusted, and risperidone was switched to haloperidol 5 mg PO twice daily. He also received benztropine 0.5 mg PO twice daily for side effect prevention. A few days later, our patient became more cooperative with the treatment team, though he remained guarded, demonstrated cognitive distortions, and lacked insight into his illness.

One week after his admission, he was less irritable but continued to exhibit disorganized behavior and cognitive impairments. The neurology team evaluated the patient and concluded unexplained neuroanatomical pathology involving bilateral basal ganglia calcifications, possibly leading to psychiatric symptoms, thus recommending further psychiatric management. Bitemporal volume loss and cerebral atrophy were noted by the team, although not clinically significant. He became less irritable and made efforts to build rapport with the treatment team, which he maintained until his discharge. His total inpatient stay was 16 days from admission to discharge. He was referred to continue outpatient care.

## Discussion

Our patient demonstrated cognitive and psychiatric dysfunctions due to isolated bilateral calcifications of the GPe, a working diagnosis of medically induced psychotic disorder. The patient’s symptoms can be explained by localization of CT imaging and clinical presentation for less than two weeks during each episode. The patient was diagnosed by obtaining collateral information from the patient's supervised residence and sibling regarding behavior and timelines, also ruling out major psychiatric diagnoses. In order to meet the criteria of a schizophrenia diagnosis, two of the following criteria must be met: delusions, hallucinations, or disorganized speech, abnormal psychomotor behavior, and negative symptoms for the period of at least six months. In addition, at least one of the following criteria must be present: delusions, hallucinations, and/or disorganized speech. All symptoms that follow these guidelines must be ongoing for at least six months. These disturbances cannot be due to substance or medications [7]. The patient does not meet criteria for schizophrenia as his episodic symptoms last for no more than two weeks’ duration. The patient denied both recent substance and medication use, urine drug screen was negative. Furthermore, substance-induced psychotic symptoms can be effectively ruled out as symptoms do not last for longer periods of times nor in the absence of substance use. The entertainment of schizophreniform is also ruled out as the duration of his illness is eight days or less and is consistent with on and off episodes without medical intervention. The patient has also been experiencing neuropsychiatric manifestations for 12 years as per patient and patient's sibling, thus effectively ruling out schizophreniform. Brief psychotic disorder remains a potential differential; however, the presence of cognitive symptoms that last beyond the duration of episodes raises the possibility of exploring other diseases that can cause cognitive symptoms. Of note, no associated characteristic basal ganglia motor symptoms were noted pertaining to pathology on the anterior territory of the GPe in this patient. Neuropsychiatric manifestations without observed motor impairments strongly indicate a pathology of the basal ganglia at certain loci instead of global damage. The specification of the locus in question manifests differently than other loci and provides information to support a correlation between neuroanatomical pathology with clinical manifestation.

Experiments have shown that the basal ganglia circuits can be categorized as motor, associative, and limbic. The motor circuit is involved in control of movements, the associative circuit plays a role in the control of executive functions, and the limbic circuit is in control of emotions and motivation. It is the dysfunction of these associative and limbic circuits that leads to neuropsychiatric symptoms [8]. Our patient presenting with psychosis, lack of motivation, and inability to follow instructions without motor presentation is suggestive of damage to the associative and limbic circuits. The association of the GPe with his non-motor symptoms in the absence of motor symptoms would be worth exploring as it was the only significant findings in the study.

Examples of such studies include those that have been conducted to examine the complex circuitry of the GP region targeting specific regions, leading to functionality. Experiments such as that described by Kelly and Strick have used retrograde viruses in order to trace circuitry from specific subnuclei of the GP and link them to functionality in non-human primates [4]. Upon analysis, the GP neurons can be seen to project to specific cortical areas indicating functionality. The GPi has been more thoroughly investigated; however, the few studies on the GPe have shown promising results.

**Figure 3:**
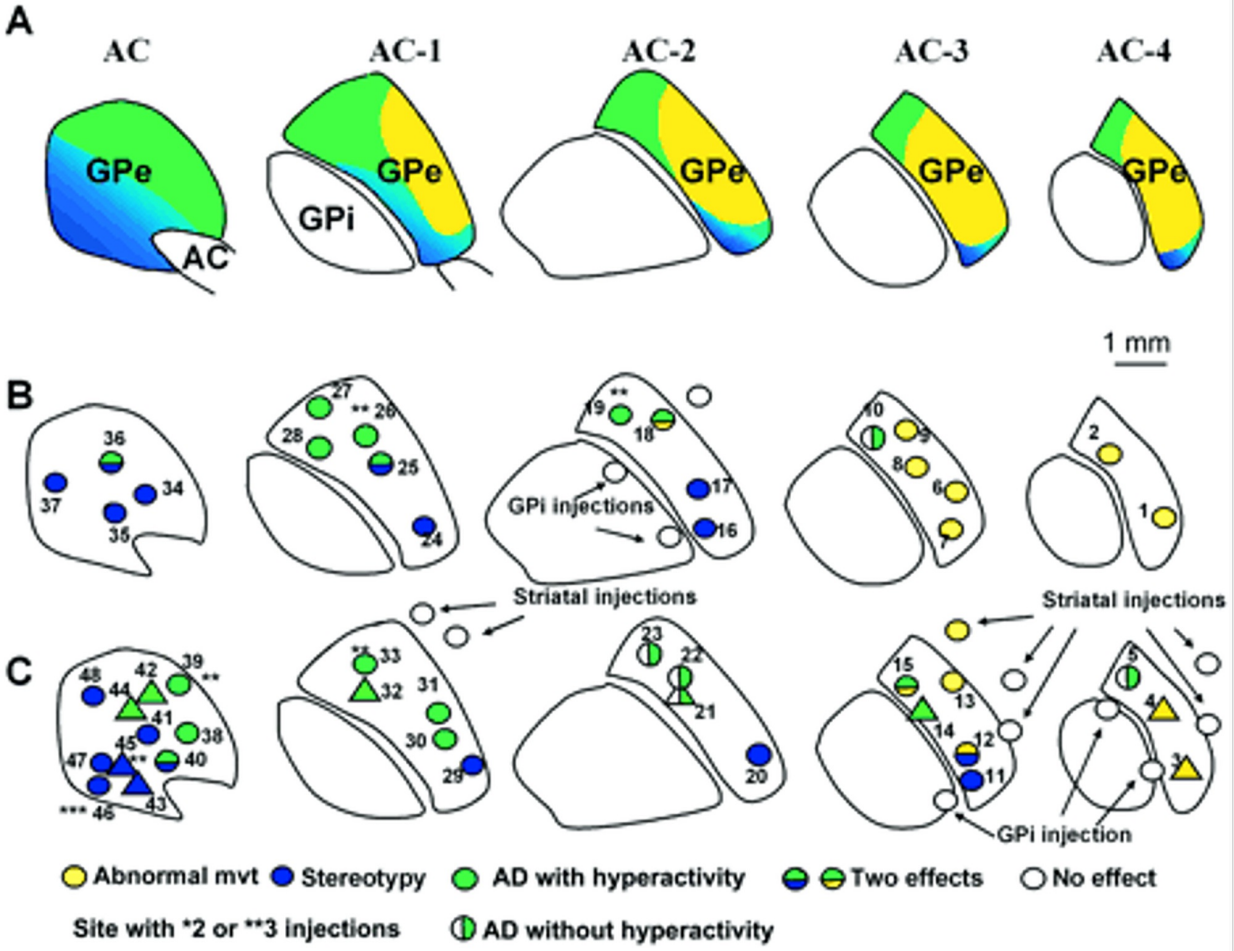
Functional territories and localization of microinjection sites in the globus pallidus externus. Source: Courtesy of Grabli et al. [10] An overview of the localization of microinjection sites and the nature of the responses obtained in non-human primate subjects. Representation of the associative (green), limbic (blue), and sensorimotor (yellow) territories within the GPe, as previously defined [11,12]. AC, anterior commissure; AD, attention deficit; GPe, globus pallidus externus; GPi, globus pallidus internus

When visualizing the GP under diffusion tensor imaging (DTI), we are able to see how similar the human GP is to that of monkeys. The regions and their projections are summarized in Table 1 and Figure 3. These similarities allow for good approximations of human basal ganglia function when testing on non-human primates. Electrophysiological studies on the basal ganglia during a variety of behaviors highlight the numerous functions. The middle to ventral portion of the posterior GP deals primarily with movement activity. This is a response to a behavioral stimulus, such as a repeated movement sequence. The movement dictated by the posteroventral area has been tested to only activate during simple movements. When accomplishing complex sequences, the GP activates both the posteroventral and anterodorsal areas. In contrast to the movement activity of the posterior GP, the anterodorsal portion has been studied to be involved in action control. This can be viewed as the desire to make an action or selection rather than the physical motor movement. The last major portion of the GP is the anteroventral portion (AVP). This has been associated with the limbic reward and inhibitory pathways. When shown a visual target indicative of a larger reward, the AVP sends an excitatory response; however, it will show an inhibitory response should the visual stimulus be something representing a small reward. This can be highlighted by looking at patients who have auto-activation deficits, induced by bi-pallidal lesions. These patients will lack all motivational drives due to the damage to the basal ganglia [9].

**Table 1:** GP segments and their projections. GP, globus pallidus

GP segment	Cortical projection
Anterodorsal portion	Association cortex
Posteroventral portion	Motor cortex
Anteroventral portion	Limbic cortex

Observation of non-motor functions independently of the motor functions of the GPe, was achieved by an experiment on three non-human primates conducted by Grabli et al. Their work demonstrated that when non-human primates were injected with bicuculline, a GABA antagonist, in the associative and limbic territories, the non-human primates all displayed abnormal behavior. Injections of bicuculline in the limbic territory of the GPe led to stereotypy, on the other hand, when the injection was localized in the associative territory, attention deficit and/or hyperactivity was observed. Of note, no movement disorders were observed as a result of these injections in all three non-human primates [10]. Localization of the microinjections along with the functional response conducted in the experiment is shown in Figure 3.

Microinjections of bicuculline were placed in a total of 48 sites throughout the GPe in monkey subjects H, A, and B while they were in their homecage. The 12 microinjections seen in Figure 3 (rows B and C: yellow shade) resulted in movements that did not belong to the usual repertoire of such animals (Table 2). The most frequent (9 out of 12 injections) consisted of irregular and sustained flexion movements. Less frequently (3 injections out of 12), the movements were more irregular and complex and were characterized by internal and external rotation or flexion and extension movements involving an entire limb [10]. Microinjection in the other 37 sites primarily localized in the anterior parts of the GPe resulted in the expected and natural behavioral acts of the animals with basic movements such as licking and biting the fingers (stereotypy); however, there was an absence of dyskinesia. The same stereotypes were observed with all three monkeys when the microinjections were performed in comparable localizations within the ventromedial part of the GPe. After the injection of bicuculline into the 13 sites prior to the return of the monkeys to their respective homecage, the stereotypies had returned with slight abnormal repetition of licking or biting of the tail or fingers, and a new abnormal behavior of licking the cage bars, which was never observed outside the effect of the bicuculline microinjections, was also noted. Another behavioral effect was a hyperactive state in which the animals expressed several behaviors from their normal repertoire. Table 2 displays a summary of the behavioral changes seen after microinjections in specific territories [6].

**Table 2:** Summary of observations made in the homecage of the three monkeys injected with bicuculline. The main categories of behaviors observed are given for each site. Circling contra., circling towards the side contralateral to the injection site; tail exam., examination of the tail by the subject; L, left; R, right

Category of Effect	Site	Non-Human Primate	Side (vol µL)	Main Behavior Witnessed
Stereotypy	29	A	L (1.5)	Licking/biting fingers, searching for food, tail exam.
Stereotypy	46	A	L (1.5)	Licking/biting fingers, licking bars, searching for food
Stereotypy	43	B	L (1.7)	Licking bars, licking/biting fingers, searching for food
Stereotypy	17	H	R (1)	Licking bars, tail exam., searching for food
Stereotypy	34	H	L (1.5)	Licking/biting fingers, searching for food
Stereotypy and hyperactivity	36	H	R (1)	Exploration, licking/biting fingers, circling contra.
Hyperactivity and dyskinesia	15	A	R (1.5)	Exploration, dyskinesia, circling contra.
Hyperactivity	31	A	R (2.3)	Exploration, licking bars, circling contra.
Hyperactivity	14	B	R (1.7)	Exploration, circling contra.
Hyperactivity	21	B	L (1.7)	Exploration, circling contra.
Hyperactivity	32	B	L (1.7)	Exploration, searching for food, circling contra.
Hyperactivity	19	H	L (1.5)	Exploration, circling contra.
Hyperactivity	27	H	L (1.5)	Exploration, circling contra.

Our case resembles the findings in non-human primates to humans with implications that the behavioral, cognitive, and mood disturbances were a result of isolated involvement of the anterior areas of the GPe. Indeed, this finding has some support in new studies by Cacciola et al. using diffusion MRI and tractography to challenge the traditional model of basal ganglia network by showing the possible existence, in the human brain, of cortico-pallidal, cortico-nigral projections. A study involving 100 healthy human subjects by Cacciola et al. concluded GP clusters showing connectivity with certain target regions, leading to functionality [6]. Cacciola et al. measured volumes and centers of gravity in order to identify GP clusters relating to these target regions. Three target regions on both the left and right hemispheres were identified in the study including sensorimotor, associative, and “other”. The results showed a significantly larger volume of GPe clusters, which targeted the associative region in comparison to sensorimotor and “other” regions. As for the GPi clusters, both the sensorimotor and associative regions were relatively equivalent. Table 3 displays the study results of volumetric clusters of the GP pertaining to target regions [6].

**Table 3:** Volumetric measurements of GP clusters at target regions. GP, globus pallidus; GPe, globus pallidus externus; GPi, globus pallidus internus

GP	Target Region	Left (mm^3^)	Right (mm^3^)
GPe	Sensorimotor	161	185
	Associative	327	284
	Other	106	114
GPi	Sensorimotor	68	67
	Associative	60	70

**Figure 4:**
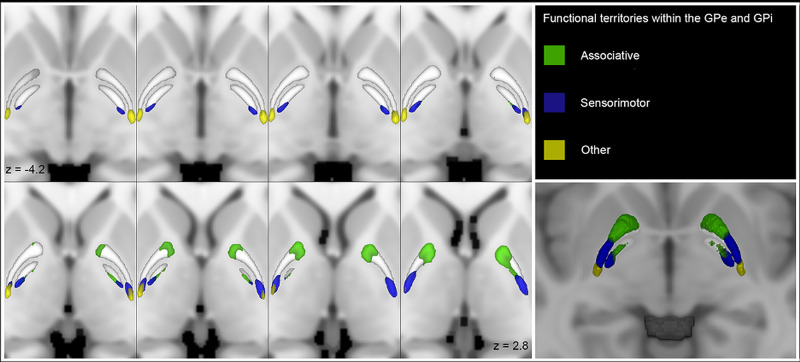
Multiple axial sections showing the GPe and GPi connectivity clusters (obtained at the 50% threshold) according to functional territories on the MNI template. Source: Courtesy of Cacciola et al. [6] The clusters have been labeled as follows: sensorimotor (blue), associative (green), and “other” (yellow). On the bottom right corner, a 3D axial section showing functional clusters within the GPe and GPi. GPe, globus pallidus externus; GPi, globus pallidus internus

Furthermore, functional differences between the GPi and GPe can be seen with their unique intricate connections with specific subregions. For example, recorded activity of the GPe neurons showed that a few neurons discharged exclusively encoding motor parameters; however, most of the discharged neurons encoded for motor, cognitive, and limbic systems. In comparison, GPi discharged neurons mainly involved in somatomotor behavior, as revealed by its functional and lobar clusters [6]. Thus, one can assume that regions associated with motor behavior in GPi and GPe are labelled “sensorimotor”. The areas labelled “associative” in GPe can explain non-motor behavioral changes with no alteration to motor behavior. This is in keeping with the findings in non-human primates and in our case. The associative, sensorimotor, and other territories are shown in Figure 4.

A review of our patient’s CT of the brain indeed shows pathology limited to the associative areas of the brain. Our case thus lends some support for the proposal by Cacciola et al. for an alternative to the traditional model of basal ganglia connections in favor of territories of “associative”, “sensorimotor”, and “other”. Such a model is more consistent with findings in non-human primates. Future investigations will need larger patient populations to evaluate the connection between the various basal ganglia pathologies with the different territories of “associative”, “sensorimotor”, and “other” and to identify if the limbic region in humans as it relates to the GP of the basal ganglia.

## Conclusions

The GPe of the basal ganglia can be divided into an anterior region and a posterior region. The anterior portion deals with associative and limbic functions, whereas the posterior deals with motor functions. Isolated pathology in the anterior region may be associated with behavioral, mood, and cognitive disturbance without motor symptoms. Future studies are needed to evaluate the findings as a new model of understanding basal ganglia pathology.
